# Health literacy demand and attitudes toward COVID-19 prevention measures among Korean American older adults and their caregivers

**DOI:** 10.1186/s12889-024-20427-7

**Published:** 2024-10-23

**Authors:** Hae-Ra Han, Ji-Young Yun, Deborah Min, Maryam Razaz

**Affiliations:** 1 School of Nursing, The Johns Hopkins University, 525 N. Wolfe Street, Baltimore, MD 21205 United States of America; 2 Bloomberg School of Public Health, The Johns Hopkins University, Baltimore, MD United States of America; 3 Grossman School of Medicine, New York University, New York, NY United States of America

**Keywords:** COVID-19, Health literacy, Korean American, Older adults, Caregivers

## Abstract

**Background:**

Health literacy has been linked to positive attitudes toward COVID-19 preventive measures among adolescents and young- or middle-aged adult populations. This study examined the relationship between health literacy and attitudes toward COVID-19 preventive measures among non-English speaking Korean American older adults and their caregivers. The study additionally investigated how sociodemographic characteristics were associated with attitudes.

**Methods:**

COVID-19 survey data was collected from potential participants for an ongoing randomized controlled trial involving both Korean American older adults and their caregivers in the Baltimore-Washington and the New York Metropolitan areas (ClinicalTrials.gov Identifier: NCT03909347). Korean American older adults with normal cognition and their caregivers were allowed to participate in the survey. We used latent profile analysis to find unique clusters of participants with a similar pattern of responses to attitudes toward COVID-19 preventive measures. Based on the analysis, we employed multinomial logistic regression to investigate how health literacy and sociodemographic characteristics were associated with the clusters.

**Results:**

We found three clusters based on participant responses to COVID-19 preventive measures—Positive, Negative, or Mixed. Health literacy was not associated with COVID-19 related attitudes in the study sample. Men were 2.37 times more likely to be categorized as Mixed than having Positive Attitudes compared to women. The odds of a person living in the New York metropolitan area being categorized as having Mixed Attitudes compared to Positive Attitudes were also 2.67 times more than for a person living in the Baltimore-Washington area.

**Conclusions:**

Differences in attitudes toward COVID-19 preventive measures were found among sociodemographic variables but not health literacy. Investigating what information channels or methods drive perception of public health information such as COVID-19 may help identify effective dissemination strategies for non-English speaking Korean older adults.

## Background

The coronavirus disease 2019 (COVID-19) pandemic has made a dramatic impact across diverse social, economic, and health dimensions of our society. As of January 2024, the COVID-19 pandemic has led to more than 6.9 million deaths worldwide (or more than 1 million deaths in the United States alone) [1, 2]. Among fast evolving scientific discoveries include myriads of health messages related to COVID-19 prevention measures, which have been disseminated through traditional (e.g., journals, newspapers, TV, radio) and non-traditional (e.g., social network services, websites) media outlets. Unfortunately, the COVID-19 pandemic has also resulted in an infodemic, or an overabundance of information, both accurate and inaccurate, about the disease [3, 4]. The infodemic has led to confusion and uncertainty about the effectiveness of various COVID-19 prevention behaviors, as well as skepticism about the severity of the virus itself, fueling risk to public health [3, 4]. To this end, there has been an increasing discussion on health literacy and the role it plays in dissemination of important health measures to curb the spread of the COVID-19 virus.

Health literacy is the “degree to which individuals have the ability to find, understand, and use information and services to inform health-related decisions and actions for themselves and others” [5]. Health literacy plays a critical role in promoting health equity and preventing the spread of infectious diseases like COVID-19. For immigrants with language barriers, such as individuals with limited English proficiency in the United States, the importance of health literacy is more pronounced as they may face additional challenges to accessing health information and navigating the healthcare system [6–9]. Nevertheless, research has rarely addressed how health literacy is associated with knowledge and attitudes toward COVID-19 preventive measures among the most vulnerable groups of people such as those with limited English proficiency.

Available research has shown that individuals with higher health literacy have more favorable attitudes toward COVID-19 prevention behaviors, such as wearing masks, social distancing, and hand hygiene [10, 11]. These studies mostly included young/middle-aged and English speaking individuals in Australia who were recruited via social media (Facebook and Instagram) [10], college students in Portugal recruited via an institutional email system (mean age = 24 years) [11], Norwegian adolescents (mean age = 17 years) recruited via email, social media platforms (Facebook and Twitter) or by an influencer (Snapchat) [12], Japanese adults (mean age = 55 years) recruited from a survey research company database [13], and Spanish university students (mean age = 22 years) with higher health literacy and more likely to engage in these behaviors [14]. None of these studies involved older adults who may have different exposure and knowledge about COVID-19 preventive measures due, in part, to their limited exposure to online resources and possible involuntary lack of digital skills (e.g., using digital devices such as a computer, tablet, or smartphone; finding and utilizing responsibly information on the internet; or communicating socially and professionally through email, text, and social media) [15].

The main purpose of this study is to examine the relationship between health literacy and attitudes toward COVID-19 prevention measures among non-English speaking Korean American older adults and their caregivers. Additionally, we investigated how sociodemographic characteristics (e.g., age, education, internet use, geographic location) were associated with attitudes toward the COVID-19 prevention measures. We used COVID-19 survey data collected from potential participants who were going through eligibility screening for an ongoing randomized controlled trial (ClinicalTrials.gov Identifier: NCT03909347). To help us better understand how Korean American older adults were being affected by COVID-19, the study team developed a survey to ask questions about possible exposure to the virus, experiences with COVID-19 testing and treatment, and how life changed as a result of COVID-19.

## Materials and methods

### Design and sample

We performed a secondary analysis of COVID-19 survey data collected from potential participants who underwent eligibility screening for PLAN (Preparing successful aging through dementia Literacy education And Navigation), a community-based randomized controlled trial (ClinicalTrials.gov Identifier: NCT03909347). Briefly, PLAN is aimed at reaching dyads inclusive of Korean older adults with probable dementia and their caregivers in the Baltimore-Washington and the New York Metropolitan areas—Maryland, Washington DC, Northern Virginia, New York, and New Jersey. The PLAN trial’s primary objective is to evaluate the effect of a study intervention on linkage to care for formal dementia evaluation for Korean American older adults with probable dementia. The intervention, delivered by trained community health workers, includes dementia literacy education and phone counseling with navigation assistance. The trial also assesses caregiver outcomes, including their dementia literacy, self-efficacy in dementia care, social support, depression, and quality of life.

The PLAN trial sample comprises dyads: specifically Korean American older adults with probable dementia and their caregivers who fulfill the inclusion criteria. Korean older adults are able to enroll if they: (1) Self-identify as first-generation Korean American; (2) Are age 65 years or older; (3) Score 1.0 + on the Clinical Dementia Rating (CDR); (4) Have a caregiver who lives in the same household or has at least weekly interactions; (5) Are able to consent or have a proxy available for consent; and (6) Provide written consent to participate in the study. Caregivers are able to enroll if they: (1) Are aged 18 years or older; and (2) Are able to read and speak Korean. The PLAN trial incorporates two screening phases, namely the Mini-Mental State Exam (MMSE) followed by the CDR. Upon meeting the MMSE criterion (< 24), dyads are invited for a CDR interview. The COVID-19 survey was conducted after the MMSE screening. Caregivers were able to participate in the survey voluntarily and without restrictions, however Korean older adults were able to participate only if the MMSE screening resulted in a score 24+, indicating normal cognition. Survey data were collected between March 1 and October 15 in 2021. Of 505 potential participants who went through eligibility screening, 220 agreed to participate in the COVID-19 survey and 175 of them completed the survey (85% older adults and 15% caregivers). After the data cleaning procedure, we had 160 participants in the analysis.

### Procedures

COVID-19 survey responses were collected by trained bilingual research staff, mostly by phone, averaging 20 minutes in length. For some participants, an online survey link was shared through email or text message due to COVID-related restrictions, resulting in 11 surveys or 6% of the total surveys completed. All survey data was collected in Korean language, and compensation was not offered for participation in this optional survey.

### Instrumentation

The COVID-19 survey was designed by the study team to gain insights into the impact of COVID-19 on the physical, emotional, and mental well-being of Korean Americans. Additionally, the survey posed questions regarding the potential exposure to the COVID-19 virus, personal experiences with testing and treatment, and the ways in which participant lives were altered due to the pandemic. The survey included several questions asking about participant sociodemographic characteristics such as age, sex, education, income, living arrangement, use of internet, use of social media, and study sites. Other study variables included health literacy and attitudes towards COVID-19 preventive measures.

Health literacy was measured using four questions addressing one’s ability to understand medical information written or verbal on a 5-point Likert scale (*1 = Never*,* 2 = Occasionally*,* 3 = Sometimes*,* 4 = Often*,* 5 = Always*) [16]. Example questions included: “How often do you have someone help you read hospital materials?” or “How often do you have problems understanding what is told to you about your medical condition?” For ease of interpretation, participant responses were recoded so that higher scores indicate greater health literacy. The four-item health literacy scale had a reliability coefficient of 0.80 in the survey sample.

We assessed attitudes towards COVID-19 preventive measures with eight questions drawn from the Understanding America Study (Module 1 – Corona Virus) [17]. Participants were asked how effective/not effective the following measures are in preventing COVID-19 infection on a five-point Likert scale *(1 = Not effective at all*,* 2 = Hardly effective*,* 3 = Somewhat effective*,* 4 = Effective*,* 5 = Very effective)*: wearing a face mask, hand washing, seeking a health care provider when feeling sick, as well as avoiding public spaces, contact with high-risk individuals, restaurants, and public transport (Note: airplanes was replaced with public transport). For reliability, Cronbach’s alpha was 0.66 in the study sample.

### Ethical considerations

The Johns Hopkins Medicine Institutional Review Board approved all procedures for this study. Verbal consent was obtained from each participant prior to conducting the COVID-19 survey. Study staff assured participants’ anonymity and confidentiality of their information and the responses to survey questions. Participants had the right to withdraw at any time.

### Analysis

SPSS version 27 for descriptive statistics and multinomial regression and R was used for this analysis. The final analysis sample included 160 participants after removing those with missing responses in some of the study variables. We employed Latent Profile Analysis (LPA) to find unique clusters of participants that have a similar response pattern regarding attitudes towards COVID-19 preventive measures [18]. We employed AIC, BIC, and entropy as fit indices to identify the adequate number of sub-groups (or clusters) in our dataset. AIC and BIC are the indices that provide information about the relative balance of model fit and parsimony and both indices are calculated using the maximum likelihood estimate. A smaller value for both indicates a better model fit [18, 19]. Entropy indicates separation between latent classes and higher entropy value represents better class separation (i.e., better model) [19]. To select an optimal LPA model, we considered not only the fit indices but also meaningfulness of the model and stability of the parameter estimates [18, 20, 21]. Profiles that contain fewer than 5% of the sample are generally regarded as spurious, which is often linked to the extraction of too many profiles [22]. Once a best fitting model was identified and selected, we used a multinomial logistic regression model to find associations of diverse sociodemographic with the clusters of preventive measures resulting from the LPA. For multinomial regression, 5% significance level was used to identify statistically significant correlates.

## Results

### Sample characteristics

Table 1 presents the major descriptive characteristics of the analysis sample. The sample were mostly in their 70s (mean = 70.3 years), female (61.0%), and well-educated (mean = 14.6 years). The majority of survey respondents resided in an individual home setting such as a single home, condo, or townhouse (69.0%). Among the respondents, 55% resided in the Baltimore-Washington metropolitan area and 45% resided in the New York metropolitan area. When asked about the use of internet and social network services (SNS), nearly three out of four participants (73.0%) reported frequent use of the internet compared to 21.0% with frequent SNS use. Most participants reported an annual income being less than *$15*,*000* (48.1%). Health literacy scores ranged from 4 to 20 with an average of 9.8. Finally, attitudes scores ranged from 21 to 40 with an average of 35.8. Of the eight items included in the attitudes towards COVID-19 preventive measures scale, *Washing your hands* had the highest mean score (4.9), while *Seeing health care provider if you feel healthy but worry that you were exposed* had the lowest mean score (3.9).

**Table 1:** Survey sample characteristics (*N* = 160)

Variable	Mean (SD) or %
Age in years (range = 40–90)	70.3 (7.5)
Female	61.0
Years of education (range = 4–23)	14.6 (3.0)
Living in single home/condo/townhome	69.0
Residing in Baltimore-Washington area	55.0
Frequent use of internet	73.0
Frequent use of SNS	21.0
Annual income	
< $10,000	23.1
$10,000 to $14,499	25.0
$15,000 to $24,999	11.9
$25,000 to $34,999	7.5
$35,000 to $49,999	13.1
$50,000 to $74,999	10.6
$75,000+	8.8
Health literacy (range = 4–20)	9.8 (4.2)
Attitudes toward COVID-19 preventive measures (range = 21–40)	35.8 (3.7)

#### Latent profile analysis

Table 2 describes relevant fit indices for different models using LPA. Based on the indices, the five-class model would be the best model, however, the study team was unable to resolve irregularity and meaningfulness of items clustered in each of the class when using the five-class. Additionally, one of the profiles of the five-class model had fewer than 5% of the survey sample (i.e., eight participants). Instead, the three-class model provided us with better interpretability and a clearer pattern of items in each cluster. To this end, we selected the three-class model as our final model.

**Table 2:** LPA model estimation fit indices for COVID-19 preventive measures

No Latent Class	AIC	BIC	Entropy
1	3639	3688	1
2	3445	3522	0.978
3	3364	3469	0.910
4	3306	3438	0.947
5	3252	3412	0.912

**Fig. 1:**
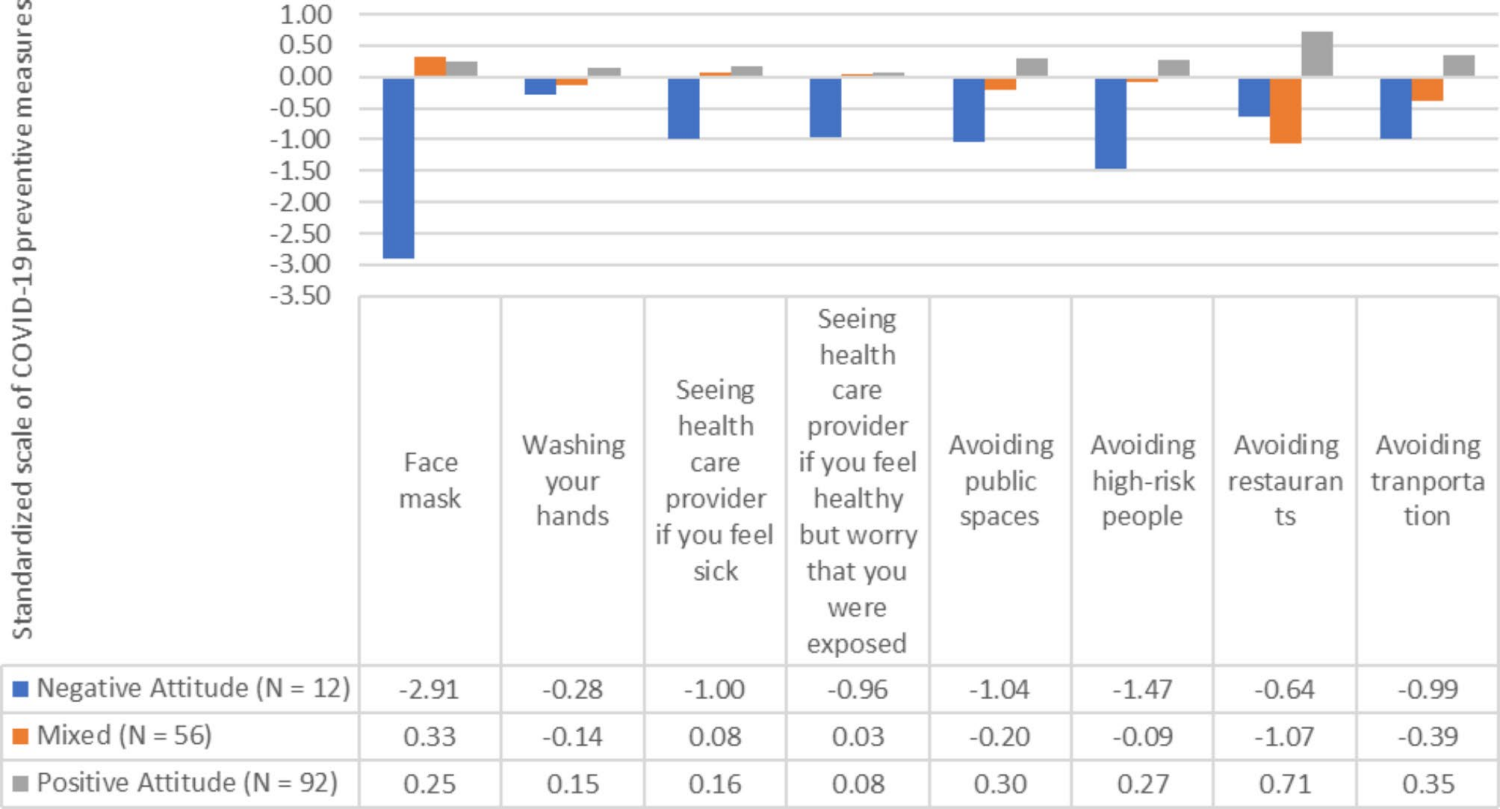
Response patterns according to the LPA classes Note: Item scores represent standardized scores using standard normal distribution (i.e., mean = 0 and SD = 1)

Using the three-class model, Fig. 1 shows response patterns of the eight COVID-19 preventive measures per class. More than half (57.5%) of the participants were classified as having “Positive Attitudes.” This group responded that all COVID-19 preventive measures were effective. 35% of the participants were classified as “Mixed.” The “Mixed” group responded that “face mask” was an effective preventive action for keeping them safe from COVID-19 and so was “seeing health provider when one was feeling sick,” while most other actions were regarded as ineffective. A small proportion (7.5%) of the participants were classified as “Negative Attitudes.” As shown in the figure, the responses of the participants in this group were below average for all eight items.

#### Multinomial logistic regression of attitudes toward COVID-19 preventive measures

Based on the finding from LPA, we used multinomial logistic regression to investigate how sociodemographic characteristics and health literacy were associated with these clusters (i.e., Negative, Mixed, and Positive Attitudes). To test the goodness-of-fit of the regression model, we employed *Pearson* and *Deviance* tests. The results of both tests indicated that the multinomial logistic model fits the data well *(p-value = 0.129 by the Pearson test; p-value = 0.940 by the deviance test).* Health literacy was not statistically significantly associated with the three clusters of attitudes. Instead, we found that gender and the geographic area variable (i.e., Baltimore-Washington versus New York metropolitan) were significantly associated with attitude towards COVID-19 preventive measures. Specifically, a male participant was more than two times (odds ratio [OR] = 2.365, 95% confidence interval [CI] = 1.095–5.108) likely to be categorized in the Mixed than in the Positive Attitudes group compared to a female participant. Similarly, the odds of a participant living in the New York metropolitan area being categorized in the Mixed compared to Positive Attitudes group was nearly three times higher (OR = 2.674, 95% CI = 1.235–5.791) than for a participant living in the Baltimore-Washington area (Table 3).

**Table 3:** Multinomial logistic regression (*N* = 160)

	Negative attitudes			Mixed attitudes		
	OR	95% CI		OR	95% CI	
		Lower	Upper		Lower	Upper
Age	0.965	0.886	1.050	1.004	0.954	1.057
Education	1.154	0.880	1.514	0.964	0.838	1.109
Income	0.849	0.592	1.217	1.024	0.842	1.245
Male	1.478	0.348	6.278	2.365 ^**^	1.095	5.108
Single home/condo/townhome	1.353	0.336	5.446	1.287	0.574	2.885
New York metropolitan	2.279	0.572	9.087	2.674^**^	1.235	5.791
Little/no use of internet	2.668	0.566	12.569	0.732	0.294	1.819
Little/no use of SNS	0.869	0.156	4.833	1.317	0.516	3.362
Health literacy	1.070	0.907	1.263	1.057	0.959	1.166

Note: *Positive Attitudes* was the reference group; Pearson and deviance statistics were not significant (*p*-value = 0.129 and 0.940 respectively); ^**^p < 0.01

## Discussion

We investigated the relationships of health literacy and sociodemographic characteristics with attitudes toward COVID-19 prevention measures among non-English speaking Korean American older adults and their caregivers. Research generally indicates positive associations among health literacy, key demographics such as education, and favorable attitudes toward COVID-19 prevention behaviors; these relationships have mainly been identified among adolescent and young- or middle-aged adult samples [10–15]. Our study is one of the first to elicit these relationships among older participants.

Health literacy was not a significant correlate of attitudes toward COVID-19 related preventive measures in the analysis sample. It is difficult to make a direct comparison of our study finding to that of previous research due to different samples used in terms of age and English language proficiency [10–15]. A possible explanation might be that standard COVID-19 precautions were widely disseminated to encourage the public to minimize the spread of infections [23], hence the impact of health literacy on the uptake of information addressing COVID-19 prevention measures might have been minimized among those such as non-English speaking individuals who are often excluded from mainstream health information dissemination. For example, COVID-19 has generated an infodemic with a huge volume of both verified and unverified information about the COVID-19 virus, preventive measures, and vaccination, presenting a major health literacy challenge [4]. Given diversified channels of information addressing COVID-19 preventive measures, our study sample may have been exposed to these measures with an overall little variance. Indeed, nearly everyone in our sample agreed that common preventive measures such as handwashing, face masks, and avoiding public spaces are effective or very effective in avoiding COVID-19 infection. A recent scoping review of 31 studies that explored COVID-19 knowledge, attitudes, and practices among Black populations [24] revealed that Black communities reported high levels of adherence to preventive measures and practices, despite the prevalent belief that they were less likely to become infected with the virus and had lower levels of COVID-19 knowledge. In the end, health literacy is considered an essential strategy to reduce susceptibility to health-related misinformation [25]. More research involving older adults from diverse backgrounds may help unravel the impact of health literacy on the uptake of health information.

We found that male gender and residence in New York were associated with more mixed than positive attitudes toward COVID-19 preventive measures. Male gender and residence in certain geographic area have been associated with positive attitudes toward COVID-19 preventive measures. For example, in a cross-sectional study of 675 adults in New York and San Francisco, male gender and residence in California were identified as correlates of positive attitude [26]. The mechanism behind the relationship between gender and attitudes regarding COVID-19 measures is not completely clear. Our finding of people in New York reporting less positive attitudes than their counterparts in Baltimore-Washington area may imply different exposures to regional mass information campaigns and experiences regarding the COVID-19 epidemic. We did not monitor particular public health initiatives or events which might have contributed to the attitudes toward COVID-19 measures in the study sample. Nevertheless, a recent geospatial analysis of COVID-19 related tweets [27] revealed that New York and California had similar numbers of misinformation tweets per 1,000 residents, when in fact New York had a higher rate of cases and hospitalizations.

Social media has been reported as a main source of COVID-19 related information across diverse populations [28–30]. We found that the use of social media was not a significant correlate of attitudes toward COVID-19 preventive measures in the analysis sample. One plausible explanation is that prior research addressing social media has predominantly involved younger populations. For example, a recent survey [30] revealed that social media served as important information sources for college students in China (*N* = 1,353) as they reported high reliance on social media for COVID-19 updates which was positively associated with higher perceived importance of preventive measures. In contrast, only about one of five Korean American older adults in our analysis sample (21%) reported using SNS fairly often or very often. Despite older adults increasingly using social media, the relationship between social media use and diverse health attitudes and behaviors among older adults is not well understood [31]. Future research is warranted to elicit the scope of social media use activities among older adults and how these activities may influence a range of attitudes and well-being outcomes.

### Limitations

Our study findings should be interpreted with consideration of several limitations. First, the survey was administered in the earlier period of the COVID-19 pandemic when broad nationwide restrictions were applied, and most research activities were conducted virtually. It is likely that most survey respondents were those who were able to follow our instructions to download Zoom or were already using it. Indeed, our study sample had high levels of education and a generally high level of health literacy, limiting the generalizability of findings. Another limitation is that, at the time of our survey, there was no effective COVID-19 treatment. In our analysis sample, the item, “seeing a health provider for actual or suspected exposure” had the lowest score out of all the COVID-19 preventive measures included in the survey. The result is likely an artifact of the timing of the survey as it was completed before more effective COVID-19 treatment methods were made available. It may very well have been the case that the survey responders felt that seeing a health provider would result in any effective treatment and hence most might have recognized it as not so effective as the other preventive measures included. Additionally, we used a convenience sample among non-English speaking Korean American older adults and their caregivers in the Baltimore-Washington and New York metropolitan areas, thus our findings may not be generalizable to non-English speaking Korean American older adults and their caregivers in other geographic regions. Finally, due to the cross-sectional nature of this study, we were unable to establish a causal relationship between health literacy, sociodemographic characteristics, and attitudes toward COVID-19 prevention measures, for which future research is needed for better exploration such as through longitudinal studies.

## Conclusions

This study showed that while the majority of Korean immigrant older adults in the analysis sample had positive attitudes toward COVID-19 preventive measures, more than a third had mixed attitudes with an additional small proportion having negative attitudes. We found that male gender and living in New York (as opposed to Baltimore-Washington) were associated with having mixed than positive attitudes. We did not find any association between health literacy and attitudes toward COVID-19 preventive measures. Our findings seem to highlight the COVID-19 related infodemic [4] through diverse channels in facing this global public health threat and suggest the importance of addressing misinformation amidst the proliferation of COVID-19 information.

## Data Availability

The datasets generated and/or analyzed during the current study are available from the corresponding author on reasonable request.

## Abbreviations

CDR: Clinical Dementia Rating
CI: Confidence interval
COVID-19: Coronavirus disease 2019
LPA: Latent Profile Analysis
MMSE: Mini-Mental State Exam
OR: Odds ratio
PLAN: Preparing successful aging through dementia Literacy education And Navigation
SNS: Social network services
